# Bacterial Bloodstream Infections in Neonates in a Developing Country

**DOI:** 10.5402/2012/508512

**Published:** 2012-08-05

**Authors:** Daynia E. Ballot, Trusha Nana, Charlotte Sriruttan, Peter A. Cooper

**Affiliations:** ^1^Department of Paediatrics and Child Health, University of the Witwatersrand, PO Wits, Johannesburg 2050, South Africa; ^2^Department of Clinical Microbiology and Infectious Diseases, National Health Laboratory Services, P.O. Box 1038, Johannesburg 2000, South Africa

## Abstract

*Background*. Ongoing surveillance of antimicrobial sensitivity patterns of bacteria isolated in bloodstream infections guides empiric antibiotic therapy in neonatal sepsis. *Methods*. Sensitivity profiles of neonatal bacterial bloodstream infections in a tertiary hospital were reviewed between 01/06/2009 and 30/06/2010 . *Results*. There were 246 episodes of bloodstream infection in 181 individuals—(14.06 episodes in10.35 patients/1000 patient days or 14.4 episodes in 10.6 babies/1000 live births. The majority were (93.5%) were late onset and most (54.9%) were gram positive. There were 2.28 sepsis-related deaths /1000 patient days or 2.3/1000 live births. Death was significantly associated with gram-negative infections (*P* < 0.001), multiple gestation (*P* < 0.001), shock (*P* = 0.008), NEC (*P* = 0.002), and shorter duration of hospital stay (*P* < 0.001). Coagulase-negative staphylococcus was isolated in 19.1%, *K. pneumoniae* ESBL in 12.1%, and *A. baumanni* in 10.9%. *S. agalactiae* predominated in early onset sepsis. Methicillin resistance was present in 86% of CoNS and 69.5% of *S. aureus*; 46% enterococcal isolates were ampicillin resistant. The majority (65%) of *K. pneumoniae* isolates were ESBL producers. Ampicillin resistance was present in 96% of *E. coli*. *Conclusions*. Penicillin and an aminoglycoside would be suitable empiric therapy for early onset sepsis and meropenem with gentamycin or ceftazidime with amikacin for late onset sepsis.

## 1. Background

Neonates are immune-compromised individuals who are prone to infection. Neonatal sepsis has significant morbidity and mortality and is difficult to diagnose on presentation. For this reason, those with suspected sepsis are commenced on empiric antibiotic therapy until sepsis can be ruled out. Overuse of antibiotics results in the development of antimicrobial-resistant organisms (ARO).

Infection with ARO results in delay in starting effective antibiotic therapy, fewer possible treatment options and increased morbidity and mortality, with prolonged hospital stay and greater costs of hospitalisation [1]. Antibiotic stewardship, including appropriate choice and administration of antibiotics, deescalation of therapy, and a multidisciplinary team approach to managing neonatal sepsis, is recommended to limit inappropriate antibiotic use and prevent the development of ARO [1].

Empiric antibiotic therapy is based on surveillance of antimicrobial sensitivity patterns in culture isolates. Pathogens vary considerably between different neonatal units. In many hospitals, gram-positive organisms cause up to 70% of nosocomial infections in neonates [1] with coagulase-negative staphylococci (CoNS) accounting for more than half of these [2]. In developing countries, gram-negative organisms may be far more prevalent as neonatal pathogens [3], with a higher incidence of antimicrobial resistance. Pathogens also vary over time, for example, the first outbreak of multiple-drug-resistant *A. baumannii *in extremely low birth weight infants in the USA was described in 2004 [4]. In a survey over 10 years in a tertiary care neonatal unit in India, *E. aerogenes *was the most common cause of late-onset sepsis (LOS) during the first 5 years, whereas *S. aureus *became the main organism causing LOS in the latter 5 years [5]. Ongoing surveillance of microbiological isolates and their sensitivity patterns is therefore an essential part of antibiotic stewardship, in particular to guide the selection of empiric therapy.

The aim of this study was to describe bacterial isolates from blood cultures obtained from the Charlotte Maxeke Johannesburg Academic Hospital (CMJAH) neonatal unit, to review antibiotic sensitivity patterns, and to provide guidelines for empiric antibiotic therapy for neonatal sepsis in a developing country.

## 2. Subjects and Methods

This was a retrospective review of all bacterial isolates in blood cultures obtained from the neonatal unit at CMJAH between 1 June 2009 and 30 June 2010. This is a tertiary care neonatal unit which cares for both inborn and outborn neonates. A list of positive blood cultures was obtained from the National Health Laboratory Service (NHLS) Computer Data Warehouse. Information obtained included identification of the organism(s) isolated and the antibiotic sensitivity of each organism. A neonate was defined as being ≤28 days of age. Only *blood stream infections (BSIs)* were included in the audit. Babies with clinical or laboratory signs suggestive of sepsis but with negative blood cultures were excluded. Other infections, such as pneumonia, conjunctivitis, urinary tract infection, meningitis, phlebitis, skin abscess, and omphalitis, were excluded. Infections were classified as *early onset (EOS)* if the positive culture was obtained before 72 hours of life and *late onset (LOS)* if the positive blood culture was obtained after 72 hours of life. A *sepsis-related death (SRD)* was diagnosed if the baby died within 5 days of developing a BSI. If more than one pathogenic organism was isolated from a single culture this was classified as a *polymicrobial infection*.

Blood cultures were performed on babies with a history of obstetric risk factors (e.g., chorioamnionitis, prolonged rupture of membranes) or who presented with signs of suspected sepsis, such as apnoea, lethargy, temperature instability, respiratory distress, hypotension, poor feeding, glucose imbalance, and seizures. A sterile 0.5 mL blood sample was sent for culture prior to commencing antibiotic therapy, together with a full blood count and C reactive protein (CRP). The CRP was repeated after 24 hours.

During the study period, EOS was treated empirically with penicillin and amikacin. Empiric therapy for LOS was modified in consultation with the microbiology unit in response to the changing antibiograms prevalent in the unit at the time. Positive blood cultures were each reviewed with the microbiology unit and a joint decision was taken on the significance of the organism and appropriate de-escalation of antibiotic therapy was instituted. A BSI was diagnosed if a pathogenic organism was isolated, the baby was clinically ill, and there were nonspecific laboratory signs of sepsis including a raised CRP, abnormal white cell count, or platelet count. Certain organisms, including *Micrococcus species, Bacillus species, *and *Corynebacterium species, *were classified as contaminants. CoNS were regarded as contaminants if the baby was clinically well, the CRP was normal, and there were no indwelling central catheters. Organisms classified as contaminants were excluded from the analysis. If the same organism was isolated from the same patient within 7 days, this was considered to be a single episode of BSI.

Limited clinical and demographic information on each patient was obtained from a neonatal computer database. This included obstetric risk factors for infection, antenatal care, use of antenatal corticosteroids, outcome, duration of hospitalization, gender, birth weight, gestational age, place of birth, birth asphyxia, HIV exposure, nasal continuous positive airways pressure (NCPAP), mechanical ventilation, necrotizing enterocolitis (NEC) grade 2 and 3 [6], surgery, and intraventricular haemorrhage (IVH) grade 3 and 4 [7]. Each pathogenic organism isolated was considered as a separate episode of BSI—if 2 pathogenic organisms were isolated from a single patient, that patient's clinical information would be entered for each separate organism. Variables were entered as yes, no, or unknown. Information regarding total patient days and total live births during the study period was obtained from admission and labour ward statistics. The live births included those from Hillbrow Community Health Centre and Alexandra Clinic.

Statistical analysis was done using SPSS version 19 (IBM). Categorical variables were described as frequencies and percentages and compared using Chi-square analysis. Fisher's exact test was used in those instances where the anticipated size of a cell was below 5. If normally distributed, continuous variables were described as mean and 95% confidence intervals and means were compared using the unpaired *t*-test. If the distribution was not normal, the data was described using median and interquartile range and compared using Mann-Whitney *U* analysis. All data was compared considering SRD, EOS, and Gram stain as outcome variables. Significant differences are reported.

The study was approved by the Human Research Ethics Committee of the University of the Witwatersrand.

## 3. Results

There were 246 episodes of BSI in 181 individuals. Details of the number of episodes of BSI and polymicrobial infection per individual patient are shown in Table 1. There were 14.06 episodes of BSI in 10.35 patients per 1000 patient days, calculated on 17485 total patient days. The total number of live births was 17 039. There were thus 14.4 episodes of BSI in 10.5 babies (0.93 episodes of EOS and 13.5 episodes of LOS) per 1000 live births. Gram-positive organisms were isolated in 135 cases (54.9%) and gram-negative ones in 111 (45.1%). EOS occurred in 16 (6.5%) cases of BSI, while 230 (93.5%) had LOS.

## 4. Mortality

SRD occurred in 40 of 181 individuals (22.1%), corresponding to in 46 episodes of BSI (17.9%), because certain babies had more than one BSI. Mortality rates in the EOS and LOS groups were not significantly different—1/16 (6.3%) as compared to 45/230 (19.6%) (*P* = 0.319). There were 2.28 SRD per 1000 patient days, 2.3 SRD per 1000 live births. Mortality was significantly greater in gram-negative infections 32/111 (28.8%) as compared to gram-positive infections 14/135 (10.4%) (*P* < 0.001 Pearson's Chi-square 13.652). Polymicrobial infections were not significantly associated with SRD—12/46 (26.1%) versus 38/200 (19%). (*P* = 0.281 Pearson's Chi-Square 1.160). Babies with single, rather than recurrent, infections were significantly more likely to die—39/46 (84. 8%) versus 133/200 (66. 5%) (Chi square 5.943 *P* = 0.015).

## 5. Clinical Associations

Clinical information was available for 140 patients (77.3%) corresponding to 195 episodes of BSI and 32 SRD. The median birthweight was 1200 grams (IQR 613 grams), mean gestational age 30.79 weeks (95% CI 30.12–31.46), and mean duration of hospital stay was 32.36 days (95% CI 29.12–35.6 SD 22.42). Clinical information is presented in Table 2.

Multiple gestation was significantly related to SRD—11/32 (34.3%) versus 14/163 (8.5%) (Chi-square 16,025 *P* < 0.001). Shock occurred more frequently in SRD—4/32 (6.7%) versus 2/163 (1.2%) (Chi square 11.86 *P* = 0.008), and SRD was significantly associated with NEC—9/163 (5.5%) versus 8/32 (25%) (Chi square 12.75 *P* = 0.002). The duration of hospital stay was significantly shorter in cases of SRD—11.78 days (SD 10.9) compared to 37.28 days (SD 22.03) (*P* < 0.001). The median birth weight of babies that died was 1045 grams (IQR 700 grams) as compared to 1200 grams (IQR 565) in survivors. This was not statistically different (*P* = 0.08). All other clinical parameters were not significantly associated with SRD.

Babies with EOS were significantly more likely to have a history of obstetric risk factors as opposed to those with LOS—5/12 (41.6%) versus 21/183 (11. 47%) (Chi Square = 9.8; *P* = 0.02). Also, babies with EOS were significantly heavier at birth (median 2.82 kg (IQR 1.97) versus median 1.2 kg (IQR 0.52) *P* = 0.002) and had a shorter duration of hospitalisation (11.5 days (SD 11.8) versus 34.3 days (SD 22.5) *P* = 0.006) than those with LOS.

## 6. Microbiology

Details of microbiology isolates are shown in Table 3. The most common isolate overall was CoNS in 47/246 (19. 1%), followed by *K. pneumoniae *ESBL in 30/246 (12.1%) and *A. baumannii *27/246 (10.9%) cases. Gram-positive infections predominated in EOS, with *S. agalactiae *being the most common pathogen. Both gram-positive and gram-negative infections were prevalent in LOS, with CoNS isolated most frequently. Gram-negative infections were significantly more frequent in multiple gestations 17/87 (19.5) compared to 8/108 (7.4) (Chi square 7.036; *P* = 0.03) and were associated with the use of antenatal corticosteroids 25/87 (28.7%) compared to 15/108 (13.8) (Chi square = 8.84; *P* = 0.031). Together *E. coli *and *K. pneumoniae *accounted for half of the SRD.

Antimicrobial resistance was common; 86% of CoNS and 69.5% of *S. aureus *were methicillin resistant. Eleven of the 24 enterococcal isolates were *E. faecium*, resistant to ampicillin. No vancomycin resistance was noted in the gram-positive organisms. Sixty-five percent of the *K. pneumoniae *isolates were ESBL producers, and 94% of these were susceptible to amikacin and 83% to ciprofloxacin. Of the 23 *E. coli*'s cultured, all but one was resistant to ampicillin. All the *E. coli*'s tested susceptible to the 3rd-generation cephalosporins and the aminoglycosides. No carbapenem resistance was detected in the Enterobacteriaceae. Ceftazidime showed the greatest activity against *A. baumannii *(96% susceptibility), followed by ciprofloxacin (89%) and gentamycin (85%).

## 7. Discussion

The choice of empiric antibiotic therapy in neonatal sepsis presents a problem. Bacterial isolates and sensitivity patterns in the current study are considerably different from those obtained in a similar review in the same unit in 2002/3 audit [8], which is in keeping with experience in other neonatal units [5]. The proportion of EOS and LOS remained similar. However, in the initial audit there were no cases of *S. agalactiae *and gram-negative organisms dominated EOS; in the present study *S. agalactiae *was the predominant isolate in EOS. A variable incidence of *S. agalactiae *has been noted in previous studies [9]. CoNS remained the most common isolate, but the proportion of CoNS declined from 49.6% of LOS in 2002/3 to 18.6% in 2009/10. It is important to note the significant increase in ESBL *K. pneumoniae, E. faecalis, *and *E. faecium, *as well as the emergence of *A. baumannii *as a significant pathogen. This increase in gram-negative pathogens is consistent with other reports from developing countries [1, 5].

Guidelines for empiric antibiotic treatment in this unit are informed by ongoing surveillance of the spectrum of bacteria causing neonatal sepsis and their antibiotic resistance profiles. Clinicians and microbiologists review sensitivity profiles and adjust empiric antibiotic therapy accordingly. Based on the bacterial profile in the present study, suitable empiric antibiotic therapy for EOS would be penicillin plus an aminoglycoside. Penicillin will provide adequate cover for *S. agalactiae*. The addition of an aminoglycoside to the penicillin broadens the antimicrobial cover and provides synergy. Both gram-positive and gram-negative empiric cover is required for suspected LOS. Vancomycin will provide cover for CoNS, MRSA, and *E. faecium*. The clinical significance of CoNS in sepsis is difficult to assess, and the SRDs for CoNS in this audit were 7% (3/43). However, SRDs for MRSA and *E. faecium* were 18% each (3/16 for MRSA and 2/11 for *E. faecium*). Due to the differing antibiograms for the *K. pneumoniae *ESBLs and the *A. baumannii*'s, a single Gram-negative antimicrobial agent will not provide optimal empiric cover. In critically ill neonates meropenem plus either gentamycin or ciprofloxacin is indicated. Alternatively, the combination of ceftazidime and amikacin will provide cover for the majority of the Enterobacteriaceae and non-fermenters. Once the offending pathogen is known, de-escalation of treatment is imperative. Ongoing surveillance of neonatal sepsis is essential in monitoring trends in infection, ensuring up-to-date appropriate antibiotic therapy, early outbreak detection, and institution of preventative measures. Continuous infection control awareness is vital in light of the predominance of LOS due to ARO like *K. pneumoniae *ESBL and MDR *A. baumannii *in this unit.

The rate of neonatal BSI in the CMJAH unit is comparable to that reported from other developing countries but is much higher than that in developed countries. A neonatal unit in the Republic of Korea had 15.1 infection in 10.2 neonates per 1000 patient days [10], whereas the rate of neonatal sepsis in developed countries is reported to be between 5 and 6 per 1000 patient days reported from developed countries [11, 12]. Note that there is a great variation in rates of nosocomial infection between different neonatal units, even in developed countries [11].

The mortality rate for LOS in the CMJAH neonatal unit remained fairly constant at around 20%, compared to the previous audit in the unit und 20% [8], while the CFR for EOS declined from 40% to 6.25%. This most likely corresponds to the decline in gram-negative pathogens in EOS between the two periods. In both time periods, mortality was significantly associated with gram-negative isolates, which is in keeping with other reports [13, 14]. Certain risk factors for sepsis, including birthweight [3, 8, 14], NCPAP [15], mechanical ventilation [16], and exposure to HIV, did not increase the risk of death in this study. SRD was associated with multiple gestation. Shock and NEC were risk factors for SRD, suggesting that those who died had more severe illness. Death generally occurred at a relatively young chronological age; survivors were more likely to have prolonged hospitalisation and recurrent BSI. Polymicrobial infection was anticipated to be a risk for SRD, but this was not so, which is in keeping with other reports [17].

## 8. Conclusions

Bacterial BSIs are an important problem in the CMJAH neonatal unit and AROs are common. There has been a significant change in pathogens with an increase in gram-negative organisms, particularly the emergence of *A. baumannii. *Based on the sensitivity profiles of bacteria in the present study, suitable empiric antibiotic therapy for EOS would be penicillin with an aminoglycoside while LOS would be covered by meropenem and gentamycin or ceftazidime and amikacin. It is, however, essential to have ongoing antimicrobial surveillance and strict adherence to infection control measures, especially hand washing, as part of antibiotic stewardship to limit AR.

## Figures and Tables

**Table 1:** Episodes of BSI and polymicrobial infections per individual patient.

Number of episodes of BSI	Total patients	Those with polymicrobial infection
Single	150	18
Two	25	5
Three	5	2
Four	1	0

Total	181	25 (13.8%)

**Table 2:** Clinical data.

Category	Number	Percentage
Female	109	55.9
Multiple gestation	25	12.8
Attended antenatal care	107	54.7
Inborn	139	71.3
HIV exposed	50	25.6
Antenatal corticosteroids	40	20.5
Obstetric risk	26	13.3
Perinatal asphyxia	41	21
Nasal CPAP	43	22.1
Mechanical ventilation	75	38.5
Surgery	24	12.3
Necrotising enterocolitis	17	8.7
Intraventricular haemorrhage (Grade 3 or 4)	16	8.2

**Table 3:** Bacterial isolates and mortality.

Organism	Total	EOS (death)	LOS (death)
Gram positive			
Staphylococci			
Coagulase negative staphylococci	62	4 (0)	58 (4)
Methicillin-resistant *Staphylococcus aureus *	16	0 (0)	16 (3)
Methicillin-sensitive *Staphylococcus aureus *	7	0 (0)	7 (2)
Enterococci			
*Enterococcus faecalis *	13	1 (1)	12 (0)
*Enterococcus faecium *	11	0 (0)	11 (2)
Streptococci			
*Streptococcus agalactiae *	10	7 (0)	3 (1)
Viridans streptococci	15	3 (0)	12 (1)
Gram negative			
Enterobacteriaceae			
*Citrobacter freundii *	1	0 (0)	1 (1)
*Enterobacter aerogenes *	1	0 (0)	1 (0)
*Enterobacter cloacae *	4	0 (0)	4 (1)
*Escherichia coli *	23	0 (0)	23 (11)
*Klebsiella oxytoca *	1	0 (0)	1 (1)
*Klebsiella pneumoniae *	12	0 (0)	12 (4)
*Klebsiella pneumonia* (ESBL)	34	1 (0)	33 (8)
*Proteus mirabilis *	1	0 (0)	1 (0)
Non-fermenters			
*Pseudomonas aeruginosa *	4	0 (0)	4 (1)
*Acinetobacter baumannii *	27	0 (0)	27 (4)
*Acinetobacter spp. *	2	0 (0)	2 (1)
*Stenotrophomonas maltophilia *	1	0 (0)	1 (0)
Other			
*Aeromonas caviae *	1	0 (0)	1 (0)

Total	246	16 (1)	230 (45)

## List of Abbreviations

AR: Antibiotic resistance
ARO: Antibiotic resistant organisms
BSI: Bloodstream infections
CFR: Case fatality rate
CMJAH: Charlotte Maxeke Johannesburg Academic Hospital
CoNS: Coagulase negative staphylococci
CRP: C reactive protein
EOS: Early-onset sepsis
ESBL: Extended beta Lactamase
IVH: Intraventricular haemorrhage
LOS: Late-onset sepsis
MDR: Multi drug resistant
MRSA: Methicillin-resistant
NCPAP: Nasal continuous positive airways pressure
NEC: Necrotising enterocolitis
NHLS: National Health Laboratory Service
SRD: Sepsis-related deaths.
